# Recall patterns and risk of primary liver cancer for subcentimeter ultrasound liver observations: a multicenter study

**DOI:** 10.1097/HC9.0000000000000073

**Published:** 2023-03-07

**Authors:** Amit G. Singal, T. Tara Ghaziani, Neil Mehta, Kali Zhou, Lauren T. Grinspan, Jihane N. Benhammou, Andrew M. Moon, Ju Dong Yang, Reena Salgia, Anjana Pillai, Elizabeth Zheng, Nicole E Rich, Purva Gopal, Prasun Jalal, Elizabeth Verna, Sruthi Yekkaluri, Samuel Phen, Jonathan Melendez-Torres, Omar Alshuwaykh, Hailey Choi, Kevin Junus, John Grady, Michael Song, Emily A. Leven, Jung Yum, Vrushab Gowda, Manaf Alsudaney, Perla Hernandez, Nirmal Desai, Neehar D. Parikh

**Affiliations:** 1 Department of Internal Medicine, UT Southwestern Medical Center, Dallas, Texas, USA; 2 Department of Internal Medicine, Stanford University, Palo Alto, California, USA; 3 Department of Internal Medicine, University of California San Francisco, San Francisco, California, USA; 4 Department of Internal Medicine, University of Southern California, Los Angeles, California, USA; 5 Department of Internal Medicine, Icahn School of Medicine at Mount Sinai, New York, New York, USA; 6 Department of Internal Medicine, University of California Los Angeles, Los Angeles, California, USA; 7 Department of Internal Medicine, University of North Carolina, Chapel Hill, North Carolina, USA; 8 Department of Internal Medicine, Cedar Sinai Medical Center, Los Angeles, California, USA; 9 Department of Internal Medicine, Henry Ford Medical Center, Detroit, Michigan, USA; 10 Division of Gastroenterology, Hepatology, and Nutrition, University of Chicago, Chicago, Illinois, USA; 11 Department of Internal Medicine, Columbia University, New York, New York, USA; 12 Department of Pathology, UT Southwestern Medical Center, Dallas, Texas, USA; 13 Department of Internal Medicine, Baylor College of Medicine, Houston, Texas, USA; 14 Department of Internal Medicine, University of Michigan, Ann Arbor, Michigan, USA

## Abstract

Patients with cirrhosis and subcentimeter lesions on liver ultrasound are recommended to undergo short-interval follow-up ultrasound because of the presumed low risk of primary liver cancer (PLC).

**Aims:**

The aim of this study is to characterize recall patterns and risk of PLC in patients with subcentimeter liver lesions on ultrasound.

**Methods:**

We conducted a multicenter retrospective cohort study among patients with cirrhosis or chronic hepatitis B infection who had subcentimeter ultrasound lesions between January 2017 and December 2019. We excluded patients with a history of PLC or concomitant lesions ≥1 cm in diameter. We used Kaplan Meier and multivariable Cox regression analyses to characterize time-to-PLC and factors associated with PLC, respectively.

**Results:**

Of 746 eligible patients, most (66.0%) had a single observation, and the median diameter was 0.7 cm (interquartile range: 0.5–0.8 cm). Recall strategies varied, with only 27.8% of patients undergoing guideline-concordant ultrasound within 3–6 months. Over a median follow-up of 26 months, 42 patients developed PLC (39 HCC and 3 cholangiocarcinoma), yielding an incidence of 25.7 cases (95% CI, 6.2–47.0) per 1000 person-years, with 3.9% and 6.7% developing PLC at 2 and 3 years, respectively. Factors associated with time-to-PLC were baseline alpha-fetoprotein >10 ng/mL (HR: 4.01, 95% CI, 1.85–8.71), platelet count ≤150 (HR: 4.90, 95% CI, 1.95–12.28), and Child-Pugh B cirrhosis (vs. Child-Pugh A: HR: 2.54, 95% CI, 1.27–5.08).

**Conclusions:**

Recall patterns for patients with subcentimeter liver lesions on ultrasound varied widely. The low risk of PLC in these patients supports short-interval ultrasound in 3–6 months, although diagnostic CT/MRI may be warranted for high-risk subgroups such as those with elevated alpha-fetoprotein levels.

## Graphical abstract

**Figure:**
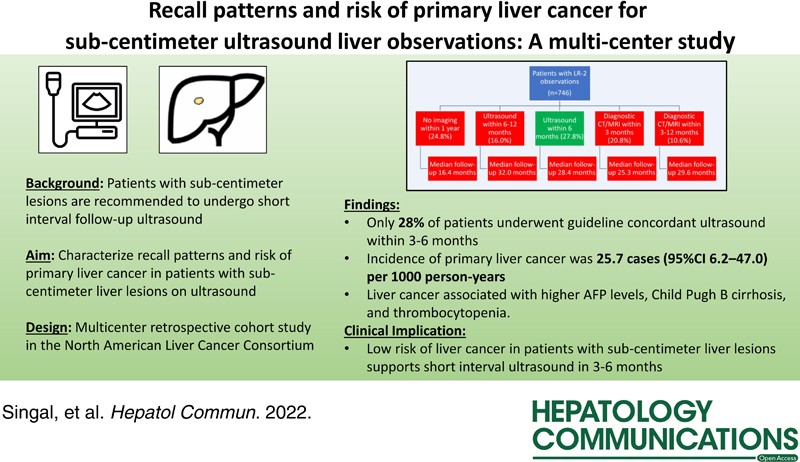

## INTRODUCTION

Patients with cirrhosis are at high risk of developing HCC, with an annual incidence of ~1%–2% per year.^1^ HCC is a leading cause of death in those with compensated cirrhosis, although prognosis highly varies by tumor stage at diagnosis. Patients with early-stage HCC can achieve 5-year survival exceeding 60% if they are eligible for liver transplantation, surgical resection, or local ablative therapy, whereas patients with more advanced tumor burden have a median survival of 2–3 years.^2^ Therefore, professional society guidelines from the American Association for the Study of Liver Diseases (AASLD) and European Association for the Study of the Liver recommend HCC surveillance in at-risk patients, including those with cirrhosis from any etiology or subgroups with noncirrhotic chronic hepatitis B infection.^3^,^4^ Several case-control and cohort studies have demonstrated that HCC surveillance is associated with significantly improved clinical outcomes, including early tumor detection and overall survival.^5^,^6^

Surveillance is performed using a semiannual abdominal ultrasound and a serum biomarker, alpha-fetoprotein (AFP), although this is one step in the larger screening continuum, which also requires timely diagnostic evaluation in those with abnormal surveillance results.^7^ Similar to the Liver Imaging and Reporting and Data System (LI-RADS) for CT and MRI findings, the American College of Radiology has proposed a classification system for ultrasound visualization and findings.^8^,^9^ The AASLD has recommended recall strategies based on ultrasound findings. Patients with liver lesions ≥1 cm (US LI-RADS 3) and those with AFP ≥20 ng/mL are recommended to undergo diagnostic multiphase CT or dynamic contrast-enhanced MRI, given a high risk of HCC.^3^,^4^ In contrast, patients with liver lesions <1 cm in maximum diameter (US LI-RADS 2) are recommended to undergo short-interval ultrasound within 3–6 months. This latter recommendation is largely based on historical studies suggesting a low risk of primary liver cancer (PLC) in patients with subcentimeter lesions.^10^–^14^ However, most studies evaluating the natural history of subcentimeter liver lesions are limited by small sample sizes, included a majority of patients having active viral hepatitis, and predated current HCC diagnostic criteria, highlighting a need for data from a contemporary cohort of patients.

Despite the guideline recommendations for ultrasound-based follow-up, there has been an increasing utilization of CT or MR imaging in clinical practice considering ultrasound’s suboptimal sensitivity, particularly in obese patients and those with nonviral liver disease.^15^,^16^ Therefore, there is also a need to better understand practice patterns for patients with subcentimeter lesions, as this informs the risk of surveillance harms and the cost-effectiveness of surveillance programs.^17^

To address these gaps, we conducted a multicenter cohort study to characterize the risk of PLC and variation in surveillance practice patterns in patients with subcentimeter liver lesions on ultrasound.

## METHODS

### Study population

We conducted a retrospective cohort study among adult patients with cirrhosis from 12 US health systems in the North American Liver Cancer Consortium.^18^,^19^ All sites were academic tertiary care referral centers with associated liver transplant programs, although 1 site had an associated safety-net health system. We included patients with cirrhosis who had at least 1 subcentimeter liver lesion between January 2017 and December 2019. Cirrhosis diagnosis was based on (1) histology, (2) noninvasive markers of fibrosis (eg, transient or MR elastography or blood-based biomarker panels) demonstrating F4 fibrosis or (3) cirrhotic-appearing liver on imaging with signs of portal hypertension (eg, intra-abdominal varices, ascites). Individuals with coexistent liver lesions ≥1 cm or any history of PLC were excluded. This study was approved by the institutional review boards at each site.

### Data collection

Demographic, clinical, and laboratory data were collected at baseline by review of the electronic medical record. Cirrhosis etiology was classified as hepatitis C (viremic vs. post-SVR), hepatitis B, alcohol-associated liver disease, NAFLD, or other.^20^ Body mass index (BMI) was categorized according to World Health Organization classification: normal (BMI <25), overweight (BMI: 25–29.99), class I obesity (BMI: 30–34.99), class II obesity (BMI: 35–39.99), and class III obesity (BMI ≥40). Liver disease severity was assessed by the Child-Pugh class, with ascites and HE classified as none, mild or controlled, and severe or uncontrolled. Laboratory indices of interest included platelet count, aspartate transaminase, alanine transaminase, bilirubin, albumin, and INR. For multivariable models, age was dichotomized at the median value (60 y), whereas laboratory values were dichotomized based on the upper limit of normal.

Ultrasound exams at each site were performed according to LI-RADS technical recommendations.^21^ Ultrasound exams were interpreted by abdominal radiologists per routine clinical care, and findings were abstracted from radiology reports. We recorded the number, maximum diameter, and location of any liver observations on each imaging study. For those who developed PLC, we recorded the method of detection (surveillance, incidental, and diagnostic) and tumor stage.

Patients were followed per institutional standard of care from the time of index imaging until progression to PLC, death, liver transplantation, or end of follow-up (date of last available CT or MRI imaging), whichever occurred earliest. We documented the receipt and imaging findings of follow-up imaging (ultrasound, CT, or MRI) or other diagnostic evaluation (eg, receipt of liver biopsy) after the index liver observation. For patients who underwent liver transplantation, we recorded explant findings, including the presence of PLC, dysplastic nodules, or any other potential pathologic correlates of interest.

### Statistical analysis

We described variation in recall patterns after detection of the subcentimeter liver observation, including the proportions with guideline-concordant versus nonconcordant follow-up. We performed a generalized estimating equation analysis, accounting for clustering by site, to identify predictors of the most common recall strategies. Variables with *p*<0.10 in univariable analyses were retained in the multivariable models, as well as observation size and AFP level given *a priori* clinical importance. For the multivariable model, we used a significance threshold of *p*<0.05.

Our primary outcome was patient-level progression to PLC, that is, LR-5 or LR-M on follow-up CT/MRI or histological confirmation, per AASLD criteria.^3^ We used the Fine-Gray subdistribution hazards model to characterize time-to-PLC development, with liver transplantation and death as competing events. Univariable and multivariable Cox regression analyses were performed to identify the factors associated with PLC. As above, observation size, AFP level, and variables with *p*<0.10 in univariable analyses were retained in the multivariable models, which relied on a backward selection process using a significance threshold of *p*<0.05. All statistical analyses were performed using SAS 9.4.

## RESULTS

### Study cohort

The baseline characteristics of the study cohort (n=746) are presented in Table 1. The median age was 59 years, and the majority (54.0%) of the cohort was male. The cohort was diverse regarding liver disease etiology (25.7% hepatitis B, 14.9% active hepatitis C, 13.0% post-SVR, 17.4% NAFLD, and 15.1% alcohol-associated) and race/ethnicity (33.8% non-Hispanic White, 21.8% Asian, 20.0% Hispanic, and 16.6% non-Hispanic Black). Most patients had compensated liver disease (78.4% Child-Pugh A). Most patients (66.0%) had a single lesion, 11.1% had 2 lesions, and the remainder had 3+ lesions. The median lesion diameter was 0.7 cm (interquartile range: 0.5–0.8 cm), with 61.3% being >0.5 cm in diameter. Median AFP was 3.4 ng/mL (interquartile range: 2.0–6.0 ng/mL), with 11.1% of patients having an AFP of >10 ng/mL. Most patients (44.8%) had adequate ultrasound visualization, although moderate and severe visualization limitations were reported in 20.8% and 2.5%, respectively. The interpreting radiologist provided a recommendation for follow-up CT or MRI in 23.6% (n=175) of cases.

**TABLE 1:** Baseline patient characteristics

Characteristic	Frequency (n=746)
Age (y)	59.0 (49–66)
Male sex, n (%)	403 (54.0)
Body mass index	
<25	281 (37.7)
25–29.9	250 (33.5)
30–34.9	129 (17.2)
35–39.9	86 (11.5)
Race/ethnicity, n (%)	
Non-Hispanic White	252 (33.8)
Hispanic White	149 (20.0)
Non-Hispanic Black	124 (16.6)
Asian	163 (21.8)
Other/not specified	58 (7.8)
Etiology of cirrhosis, n (%)	
Viremic hepatitis C	111 (14.9)
Post-SVR hepatitis C	97 (13.0)
Hepatitis B	192 (25.7)
Alcohol-associated	113 (15.1)
NAFLD	130 (17.4)
Other	103 (13.8)
Child-Pugh class, n (%)	
A	585 (78.4)
B	120 (16.1)
C	41 (5.5)
Laboratory values	
ALT, U/L	32 (23–50)
AST, U/L	35 (25–55)
Platelet count (×10^9^/L)	152 (102–210)
AFP (ng/mL)	3.4 (2.0–6.0)
No. liver observations, n (%)	
1	490 (66.0)
2	82 (11.1)
3	170 (22.9)
Observation maximum diameter (cm)	0.7 (0.5–0.8)
Ultrasound visualization	
No or minimal limitations	334 (44.8)
Moderate limitations	155 (20.8)
Severe limitations	18 (2.4)
Missing	239 (32.0)

Abbreviations: AFP, alpha-fetoprotein; ALT, alanine transaminase; AST, aspartate transaminase; SVR, sustained virological response.

### Variation in recall procedures

**FIGURE 1:**
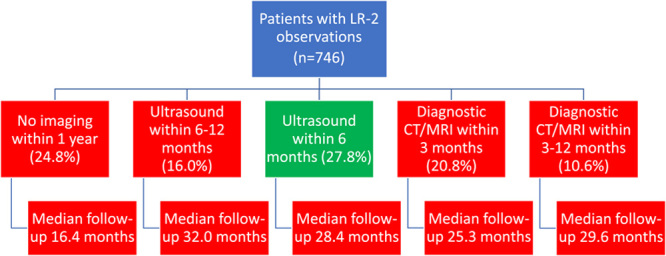
Variation in recall strategies for patients with subcentimeter liver observations on ultrasound.

Follow-up of patients with subcentimeter ultrasound lesions was variable, with only 27.8% receiving guideline-concordant ultrasound within 3–6 months, ranging from 0% to 40.3% across sites (Figure 1). There were 5 sites in which ≤10% of patients underwent ultrasound within 3–6 months and 4 sites with ≥30% of patients. The most common alternative strategies were CT/MRI within 3 months (20.8%) and ultrasound within 6–12 months (16.0%). One fourth (24.8%) of patients failed to receive repeat imaging within 1 year of index ultrasound, ranging from 9.7% to 41.7% across sites. Three sites had more than one third of patients fail to undergo repeat imaging within 1 year of the subcentimeter liver lesion detection.

Factors inversely associated with guideline-concordant follow-up, that is, ultrasound within 3–6 months, in multivariable analysis included observation size >5 mm (OR: 0.73, 95% CI, 0.57–0.93), severe visualization limitations (OR: 0.49, 95% CI, 0.37–0.64), and radiologist recommendation for CT/MRI (OR: 0.30, 95% CI, 0.15–0.59) (Table 2). Conversely, severe visualization limitations (OR: 3.36, 95% CI, 2.52–4.49) and AFP >10 ng/mL (OR: 1.47, 95% CI, 1.11–1.95) were significantly associated with increased odds of undergoing diagnostic CT/MRI within 3 months. Liver observations >5 mm (OR: 2.01, 95% CI, 0.95–4.26) and radiologist recommendation for CT/MRI (OR: 2.85, 95% CI, 0.93–8.68) were also associated with diagnostic CT/MRI within 3 months in multivariable analysis, but these did not reach statistical significance.

**TABLE 2:** Factors associated with guideline-concordant follow-up of subcentimeter liver observation

Characteristic	Univariable OR (95% CI)	Multivariable OR (95% CI)
Age (y)≤60 y	Reference	—
>60 y	1.16 (0.87–1.55)	—
Female sex	0.94 (0.70–1.27)	—
Body mass index		
<25	Reference	Reference
25–29.9	1.11 (0.65–1.88)	1.06 (0.52–2.17)
30–34.9	1.02 (0.73–1.43)	1.02 (0.69–1.53)
35–39.9	1.53 (0.92–2.54)	1.62 (0.85–3.09)
Race/ethnicity		
Non-Hispanic White	Reference	—
Hispanic White	1.41 (0.93–2.15)	—
Non-Hispanic Black	1.25 (0.76–2.07)	—
Asian	1.28 (0.67–2.43)	—
Other/not specified	0.98 (0.63–1.51)	—
Etiology of cirrhosis		
Alcohol-associated	Reference	Reference
Viremic hepatitis C	1.83 (1.08–3.12)	1.70 (0.99–2.91)
Post-SVR hepatitis C	1.24 (0.68–2.24)	1.11 (0.51–2.42)
Hepatitis B	1.08 (0.64–1.80)	1.11 (0.60–2.06)
NAFLD	1.03 (0.66–1.60)	0.96 (0.58–1.58)
Other	0.70 (0.32–1.51)	0.89 (0.35–2.21)
Child-Pugh class		
Child-Pugh A	Reference	—
Child-Pugh B	1.17 (0.93–1.48)	—
Child-Pugh C	0.74 (0.48–1.15)	—
ALT >35 U/L	1.21 (0.78–1.87)	—
AST >40 U/L	1.20 (0.84–1.70)	—
Platelet count ≤150×10^9^/L	1.25 (0.88–1.77)	—
AFP >10 ng/mL	1.43 (1.15–1.77)	1.20 (0.60–2.38)
Visualization limitations		
Minimal limitations	Reference	Reference
Moderate limitations	0.83 (0.53–1.30)	0.87 (0.50–1.52)
Severe limitations	0.48 (0.37–0.64)	0.49 (0.37–0.64)
No. observations		
1	Reference	—
2	0.64 (0.35–1.14)	—
3	0.90 (0.68–1.19)	—
Maximum diameter >5 mm	0.80 (0.66–0.98)	0.73 (0.57–0.93)
Radiologist recommendation for CT/MRI	0.26 (0.10–0.68)	0.30 (0.15–0.59)

Abbreviations: AFP, alpha-fetoprotein; ALT, alanine transaminase; AST, aspartate transaminase; SVR, sustained virological response.

### Development of PLC

**FIGURE 2:**
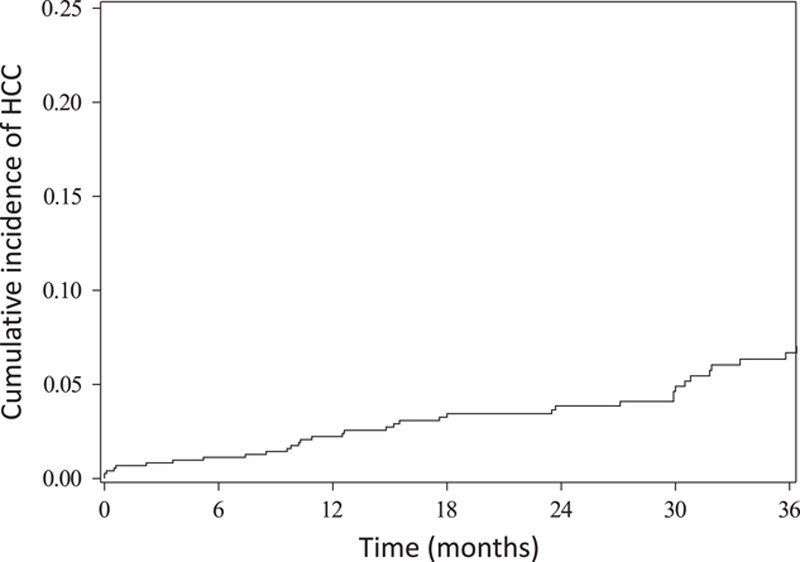
Time to primary liver cancer in patients with subcentimeter liver observations on ultrasound.

Over a median follow-up of 26 months, 57 patients died, 17 underwent liver transplant, and 42 patients developed PLC (39 HCC and 3 CCA), yielding an incidence of 25.7 PLC cases (95% CI, 6.2–47.0) per 1000 person-years (Figure 2). Cumulative incidence rates at 2, 3, and 4 years were 3.9% (95% CI, 2.5–5.6%), 6.7% (95% CI, 4.7–9.2%), and 10.1% (95% CI, 6.8–14.2%), respectively. The median time-to-PLC diagnosis was 17.8 (interquartile range: 9.6–31.9) months. Factors associated with time-to-PLC in multivariable analysis were AFP >10 ng/mL (HR: 4.01, 95% CI, 1.85–8.71), platelet count ≤150 (HR: 4.90, 95% CI, 1.95–12.28), and Child-Pugh B cirrhosis (vs. Child-Pugh A: HR: 2.54, 95% CI, 1.27–5.08) (Table 3). Incidence rates of PLC were 48.4 versus 7.5 per 1000 person-years in those with and without thrombocytopenia, 71.6 versus 22.6 per 1000 person-years in those with AFP >10 ng/mL versus ≤10 ng/mL, and 73.7 versus 17.3 per 1000 person-years in those with Child-Pugh B versus A cirrhosis. Observation size was not associated with hazards of PLC (HR: 1.35, 95% CI, 0.68–2.71), with incidence rates of 30.2 versus 18.6 per 1000 person-years for those with observations >5 mm versus ≤5 mm, respectively. Similarly, the incidence was higher among those who underwent CT/MRI within 3 months than those who underwent ultrasound within 6 months (37.1 vs. 22.7 per 1000 person-years), although this difference did not reach statistical significance (*p*=0.25).

**TABLE 3:** Factors associated with development of PLC in those with subcentimeter liver observations

Characteristic	Univariable HR (95% CI)	Multivariable HR (95% CI)
Age (y)		
≤60	Reference	—
>60	1.33 (0.73–2.41)	—
Female sex	0.80 (0.43–1.48)	—
Body mass index		
<25	Reference	—
25–29.9	1.70 (0.78–3.71)	—
30–34.9	1.71 (0.67–4.34)	—
35–39.9	2.55 (1.00–6.48)	—
Race/ethnicity		
Non-Hispanic White	Reference	—
Hispanic White	1.55 (0.73–3.26)	—
Non-Hispanic Black	1.41 (0.60–3.27)	—
Asian	0.22 (0.05–0.93)	—
Other/not specified	1.39 (0.48–4.06)	—
Etiology of cirrhosis		
Alcohol-associated	Reference	—
Viremic hepatitis C	1.13 (0.42–3.06)	—
Post-SVR hepatitis C	1.11 (0.39–3.01)	—
Hepatitis B	0.22 (0.06–0.82)	—
NAFLD	1.07 (0.41–2.83)	—
Other	1.00 (0.36–2.76)	—
Child-Pugh class		
Child-Pugh A	Reference	Reference
Child-Pugh B	3.26 (1.74–6.12)	2.54 (1.27–5.08)
Child-Pugh C	2.22 (0.65–7.63)	1.73 (0.45–6.65)
ALT >35 U/L	1.76 (0.94–3.28)	—
AST >40 U/L	2.30 (1.24–4.26)	—
Platelet count ≤150×10^9^/L	5.88 (2.47–14.02)	4.90 (1.95–12.28)
AFP >10 ng/mL	3.06 (1.46–6.45)	4.01 (1.85–8.71)
Visualization limitations		
Minimal limitations	Reference	—
Moderate limitations	1.23 (0.54–2.76)	—
Severe limitations	1.87 (0.24–14.82)	—
No. observations		
1	Reference	—
2	0.54 (0.16–1.80)	—
3	0.74 (0.34–1.59)	—
Maximum diameter >5 mm	1.66 (0.85–3.22)	1.35 (0.68–2.71)
Radiologist recommendation for CT/MRI	1.10 (0.54–2.24)	—

Abbreviations: AFP, alpha-fetoprotein; ALT, alanine transaminase; AST, aspartate transaminase; SVR, sustained virological response.

Only 15 (38.5%) of patients with HCC had Barcelona Clinic Liver Cancer stage 0/A HCC and 21 (50.0%) were within Milan Criteria at diagnosis. Two patients with CCA had metastatic disease at diagnosis, whereas 1 was found at an early stage. The proportion of early-stage PLC was 63.6% and 69.2% in patients who underwent ultrasound within 6 months (n=7/11) and MRI within 3 months (n=9/13), respectively, compared with 50% for those who underwent ultrasound within 6–12 months (n=2/4) or CT/MRI within 3–12 months (n=3/6) and 12.5% for those who had no imaging within 1 year (n=1/8). Of the 17 patients who underwent liver transplantation without PLC, 2 patients had small hemangiomas and one had a dysplastic nodule, but the others did not have any noted pathologic correlate for the ultrasound liver observation.

## DISCUSSION

In this multicenter contemporary cohort of patients with subcentimeter liver observations on abdominal ultrasound, we observed large variation in recall patterns, with less than one third undergoing guideline-concordant follow-up ultrasound in 3–6 months. This variation is highlighted by the finding that one-fifth of patients underwent diagnostic CT/MRI within 3 months, whereas one fourth failed to have any repeat imaging within 1 year. Patients had a PLC incidence of 25.7 per 1000 person-years, supporting ultrasound in 3–6 months as a guideline recommendation for this group of patients. However, diagnostic CT/MRI may be warranted in some patient subgroups with higher PLC risk, such as those with Child-Pugh B cirrhosis, clinically significant portal hypertension, or elevated AFP levels.

Guideline recommendations for short-interval ultrasound is based on 3 principles: a low short-term risk of PLC in these patients, the low sensitivity of diagnostic imaging in patients with lesions <1 cm, and sufficiently long tumor doubling times for those with HCC. However, we found only 1 in 4 patients received guideline-concordant follow-up using ultrasound within 6 months. We noted both surveillance “overuse,” with ~20% undergoing short-interval CT/MRI, and “underuse,” with >40% undergoing only intermittent surveillance. The former has implications for the enumeration of physical harms and cost-effectiveness, whereas the latter can mitigate surveillance benefits.^16^,^22^ We found radiologist recommendation for CT/MRI was associated with significantly lower odds of ultrasound within 3–6 months, highlighting this as a potential intervention target to promote guideline-concordant follow-up.

Variation in the follow-up of subcentimeter ultrasound lesions may also be related to evolving data regarding tumor doubling times and accuracy of diagnostic imaging to characterize small liver lesions. Recent studies demonstrate a median tumor doubling time of ~5–7 months, although over one fourth of patients have a rapid doubling time of <3 months.^23^,^24^ Notably, one of the most consistent correlates of rapid growth included small tumor size, likely in part related to tumor growth kinetics.^24^ Although few studies specifically examine the accuracy of diagnostic imaging for lesions <1 cm, MRI seems to have a sensitivity of 69% (95% CI, 54%–81%) for these lesions.^25^ Outside of HCC detection, diagnostic imaging can also help differentiate those patients with suspicious lesions (LR-4) versus those with indeterminate lesions (LR-3).^26^,^27^ These observations have the differential risk of developing HCC over time, and differentiating the 2 can help inform which patients are best followed by cross-sectional imaging and which patients are the sufficiently low risk that ultrasound surveillance is acceptable.

Our study directly informs the expected natural history and risk of PLC in subcentimeter liver lesions on ultrasound. Prior studies reported a wide variation in PLC risk, ranging from 15% (2 of 13 lesions) in a study by Forner et al^10^ to 69% (33 of 48 lesions) in a study by Caturelli et al.^11^ Notably, Trinchet et al^13^ found a higher proportion of subcentimeter lesions in patients undergoing quarterly ultrasound-based surveillance than semiannual surveillance; however, only 19% were confirmed as HCC at the end of the trial follow-up. In this contemporary cohort of patients, we found patients with subcentimeter liver lesions on ultrasound had a PLC Incidence of 22.3 per 1000 person-years. This incidence rate parallels that reported in broader cohorts of patients with cirrhosis, suggesting that ultrasound within 3–6 months is a reasonable strategy for these patients. Risk stratification models, using clinical risk factors such as AFP level and degree of liver dysfunction, may help identify patient subgroups who could benefit from MRI or CT imaging.^28^ More nuanced approaches incorporating radiomics or blood-based biomarkers may also be helpful to augment the accuracy of risk stratification models.^29^,^30^ A prior modeling study suggested a risk-stratified surveillance strategy among patients with cirrhosis would be cost-effective compared with a “one-size-fits-all” ultrasound-based approach.^31^ Of course, one unintended consequence of this approach would be adding health care visits and resultant indirect costs to patients.^32^,^33^ Another workflow could be same-day contrast-enhanced ultrasound, although its performance characteristics in this patient population with lesions <1cm would need to be defined.

We acknowledge several study limitations. First, there was variable follow-up among patients, given the retrospective nature of the study, which may have resulted in ascertainment bias for PLC diagnoses. Our findings should be validated using prospectively collected data from a large patient cohort with standardized imaging follow-up. Second, our study was retrospective in nature and, therefore, liable to residual confounding. For example, we identified factors associated with recall strategies but were unable to identify some potential drivers of behavior, including fear of medical malpractice litigation. Third, our study relied on reports from interpreting radiologists, which could result in measurement error given the poor interobserver reliability of ultrasound interpretation.^34^ Studies in which ultrasounds are independently reviewed by expert radiologists, with or without radiomics for lesion detection, should be considered to better characterize the natural history of subcentimeter liver lesions. Fourth, a limited number of patients in our cohort progressed to PLC, so we may have been underpowered to identify predictors of disease progression. Finally, we included multiple sites in the US, although our results, particularly those describing practice variation, may not be generalized to nonacademic settings or those outside the US. We believe these limitations are balanced by strengths of our study including the use of a large, contemporary multicenter cohort of patients and the availability of detailed clinical, laboratory, and imaging data over long-term follow-up.

In summary, we found a large variation in follow-up imaging performed in patients with cirrhosis and subcentimeter liver lesions on abdominal ultrasound. The risk of PLC in these patients supports short-interval ultrasound as a reasonable recall recommendation, although diagnostic CT/MRI imaging may be warranted in some subgroups with higher PLC risk, such as patients with more advanced cirrhosis or those with elevated AFP levels.

## References

[1] Moon AM Singal AG Tapper EB. Contemporary epidemiology of chronic liver disease and cirrhosis. Clinical Gastro and Hepatology. 2020;18:2650–66. 10.1016/j.cgh.2019.07.060 PMC7007353 31401364

[2] Reig M Forner A Rimola J Ferrer-Fàbrega J Burrel M Garcia-Criado Á. BCLC strategy for prognosis prediction and treatment recommendation: the 2022 update. J Hepatol. 2022;76:681–93. 34801630 10.1016/j.jhep.2021.11.018 PMC8866082

[3] Marrero JA Kulik LM Sirlin CB Zhu AX Finn RS Abecassis MM. Diagnosis, staging, and management of hepatocellular carcinoma: 2018 practice guidance by the American Association for the Study of Liver Diseases. Hepatology. 2018;68:723–50. 29624699 10.1002/hep.29913

[4] Galle PR Forner A Llovet JM Mazzaferro V Piscaglia F Raoul JL. European Association for the Study of the Liver. EASL clinical practice guidelines: management of hepatocellular carcinoma. J Hepatol. 2018;69:182–236. 29628281 10.1016/j.jhep.2018.03.019

[5] Singal AG Zhang E Narasimman M Rich NE Waljee AK Hoshida Y. HCC surveillance improves early detection, curative treatment receipt, and survival in patients with cirrhosis: a systematic review and meta-analysis. J Hepatology. 2022;77:128–39. 10.1016/j.jhep.2022.01.023 PMC9232881 35139400

[6] Singal AG Pillai A Tiro J. Early detection, curative treatment, and survival rates for HCC surveillance in patients with cirrhosis: a meta-analysis. PLOS Med. 2014;11:e1001624. 24691105 10.1371/journal.pmed.1001624 PMC3972088

[7] Singal AG Lok AS Feng Z Kanwal F Parikh ND. Conceptual model for the hepatocellular carcinoma screening continuum: current status and research agenda. Clin Gastroenterol Hepatol. 2022;20:9–18. 32961340 10.1016/j.cgh.2020.09.036 PMC8287785

[8] Chernyak V Fowler KJ Kamaya A Kielar AZ Elsayes KM Bashir MR. Liver imaging reporting and data system (LI-RADS) 2018: imaging of hepatocellular carcinoma (HCC) in at-risk patients. Radiology. 2018;289:816–30. 30251931 10.1148/radiol.2018181494 PMC6677371

[9] Tang A Singal AG Mitchell DG Hecht EM Fowler KJ Kulik L. Introduction to the Liver Reporting and Data System (LI-RADS) for hepatocellular carcinoma. Clin Gastroenterol Hepatol. 2019;17:1228–38. 30326302 10.1016/j.cgh.2018.10.014

[10] Forner A Vilana R Ayuso C Bianchi L Solé M Ayuso JR. Diagnosis of hepatic nodules 20 mm or smaller in cirrhosis: prospective validation of the noninvasive diagnostic criteria for hepatocellular carcinoma. Hepatology. 2008;47:97–104. 18069697 10.1002/hep.21966

[11] Caturelli E Solmi L Anti M Fusilli S Roselli P Andriulli A. Ultrasound guided fine needle biopsy of early hepatocellular carcinoma complicating liver cirrhosis: a multicentre study. Gut. 2004;53:1356–62. 15306600 10.1136/gut.2003.032359 PMC1774185

[12] Horigome H Nomura T Saso K Itoh M Joh T Ohara H. Limitations of imaging diagnosis for small hepatocellular carcinoma: comparison with histological findings. J Gastro Hep. 1999;14:559–65. 10.1046/j.1440-1746.1999.01915.x 10385065

[13] Trinchet JC Chaffaut C Bourcier V Degos F Henrion J Fontaine H. Ultrasonographic surveillance of hepatocellular carcinoma in cirrhosis: a randomized trial comparing 3- and 6-month periodicities. Hepatology. 2011;54:1987–97. 22144108 10.1002/hep.24545

[14] Tse J Shen L Bird K Yoon L Kamaya A. Outcomes of LI-RADS US-2 subthreshold observations detected on surveillance ultrasound. Am J Roentgenology. 2022;219:774–83. 10.2214/AJR.22.27812 35703411

[15] Schoenberger H Chong N Fetzer DT Rich NE Yokoo T Khatri G. Dynamic changes in ultrasound quality for hepatocellular carcinoma screening in patients with cirrhosis. Clin Gastroenterol Hepatol. 2022;20:1561–9.e4. 34119640 10.1016/j.cgh.2021.06.012 PMC8660956

[16] Chong N Schoenberger H Yekkaluri S Fetzer DT Rich NE Yokoo T. Association between ultrasound quality and test performance for HCC surveillance in patients with cirrhosis: a retrospective cohort study. Alimentary Pharmacology and Therapeutics. 2022;55:683–90. 35170052 10.1111/apt.16779

[17] Singal AG Patibandla S Obi J Fullington H Parikh ND Yopp AC. Benefits and harms of hepatocellular carcinoma surveillance in a prospective cohort of patients with cirrhosis. Clinical Gastroenterology Hepatology. 2021;19:1925–32. 32920214 10.1016/j.cgh.2020.09.014 PMC7943645

[18] Singal AG Rich NE Mehta N Branch AD Pillai A Hoteit M. Direct acting antiviral therapy for HCV infection is associated with increased survival in patients with a history of hepatocellular carcinoma. Gastroenterology. 2019;157:1253–63. 31374215 10.1053/j.gastro.2019.07.040 PMC6815711

[19] Singal AG Rich NE Mehta N Branch A Pillai A Hoteit M. Direct acting antiviral Therapy is not associated with recurrence of hepatocellular carcinoma in a multicenter North American cohort study. Gastroenterology. 2019;156:1683–92. 30660729 10.1053/j.gastro.2019.01.027 PMC6598433

[20] Hester CA Rich NE Singal AG Yopp AC. Comparative analysis of nonalcoholic steatohepatitis- versus viral hepatitis- and alcohol-related liver disease-related hepatocellular carcinoma. J National Comprehensive Cancer Network. 2019;17:322–9. 10.6004/jnccn.2018.7105 30959469

[21] American College of Radiology website. Ultrasound LI-RADS v2017. Accessed November 13, 2020. https://www. acr.org/Clinical-Resources/Reporting-and-Data-Systems/LI-RADS/Ultrasound- LI-RADS-v2017

[22] Parikh ND Singal AG Hutton DW Tapper EB. Cost effectiveness of hepatocellular carcinoma surveillance: An assessment of benefits and harms. Am J Gastro. 2020;115:1642–49. 10.14309/ajg.0000000000000715 PMC7541544 32530829

[23] Nathani P Gopal P Rich N Yopp A Yokoo T John B. Hepatocellular carcinoma tumor volume doubling time: a systematic review and meta-analysis. Gut. 2021;70:401–7. 32398224 10.1136/gutjnl-2020-321040 PMC7657990

[24] Rich NE John BV Parikh ND Rowe I Mehta N Khatri G. Hepatocellular carcinoma demonstrates heterogeneous growth patterns in a multicenter cohort of patients with cirrhosis. Hepatology. 2020;72:1654–65. 32017165 10.1002/hep.31159 PMC7398837

[25] Roberts LR Sirlin CB Zaiem F Almasri J Prokop LJ Heimbach JK. Imaging for the diagnosis of hepatocellular carcinoma: a systematic review and meta-analysis. Hepatology. 2018;67:401–21. 28859233 10.1002/hep.29487

[26] Onyirioha K Joshi S Burkholder D Yekkaluri S Parikh ND Singal AG. Clinical outcomes of patients with suspicious (LI-RADS 4) liver observations. Clin Gastroenterol Hepatol. 2022. doi:10.1016/j.cgh.2022.03.038 35413448

[27] Arvind A Joshi S Zaki T Burkholder D Parikh ND Singal AG. Risk of hepatocellular carcinoma in patients with indeterminate (LR-3) liver observations. Clin Gastroenterol Hepatol. 2022. doi:10.1016/j.cgh.2021.11.042. PMC9184301 34902571

[28] Ioannou GN Tang W Beste LA Tincopa MA Su GL Van T. Assessment of a deep learning model to predict hepatocellular carcinoma in patients with hepatitis C cirrhosis. JAMA Network Open. 2020;3:e2015626. 32870314 10.1001/jamanetworkopen.2020.15626 PMC7489819

[29] Fujiwara N Kobayashi M Fobar AJ Hoshida A Marquez CA Koneru B. A blood-based prognostic liver secretome signature and long-term hepatocellular carcinoma risk in advanced liver fibrosis. MED. 2021;2:836–50. 34318286 10.1016/j.medj.2021.03.017 PMC8312635

[30] Harding-Theobald E Louissaint J Maraj B Cuaresma E Townsend W Mendiratta-Lala M. Radiomics systematic review: radiomics for the diagnosis and prognosis of hepatocellular carcinoma. Aliment Pharmacol Ther. 2021;54:890–901. 34390014 10.1111/apt.16563 PMC8435007

[31] Goossens N Singal AG King LY Andersson KL Fuchs BC Besa C. Cost-effectiveness of risk score-stratified hepatocellular carcinoma screening in patients with cirrhosis. Clinical Translational Gastroenterology. 2017;8:e101. 28640287 10.1038/ctg.2017.26 PMC5518949

[32] Nguyen M Roberts L Engel-Nitz N Bancroft T Ozbay B Singal AG. Gaps in hepatocellular carcinoma surveillance in a nationwide cohort of insured US patients with cirrhosis. Curr Med Res Opin. 2022;38:2163-–73. 36111416 10.1080/03007995.2022.2124070

[33] Petrasek J Singal AG Rich NE. Harms of hepatocellular carcinoma surveillance. Curr Hepatol Rep. 2019;18:383–9. 33214987 10.1007/s11901-019-00488-8 PMC7673298

[34] Fetzer DT Browning T Xi Y Yokoo T Singal AG. Associations of ultrasound LI-RADS visualization score with examination-, sonographer-, and radiologist-factors: retrospective assessment in over 10,000 examinations. Am J Roentgenology. 2022;218:1010–20. 10.2214/AJR.21.26735 PMC9270853 34910539

